# Effect of Expression of Nuclear-Encoded Cytochrome C Oxidase Subunit 4 Isoforms on Metabolic Profiles of Glioma Cells

**DOI:** 10.3390/metabo12080748

**Published:** 2022-08-16

**Authors:** Claudia R. Oliva, Md Yousuf Ali, Susanne Flor, Corinne E. Griguer

**Affiliations:** 1 Free Radical & Radiation Biology Program, Department of Radiation Oncology, The University of Iowa, Iowa City, IA 52242, USA; 2 Interdisciplinary Graduate Program in Human Toxicology, Department Radiation Oncology, The University of Iowa, Iowa City, IA 52242, USA

**Keywords:** cytochrome c oxidase, metabolomics, COX4-1, glioma

## Abstract

Although often effective at treating newly diagnosed glioblastoma (GBM), increasing evidence suggests that chemo- and radiotherapy-induced alterations in tumor metabolism promote GBM recurrence and aggressiveness, as well as treatment resistance. Recent studies have demonstrated that alterations in glioma cell metabolism, induced by a switch in the isoform expression of cytochrome c oxidase subunit 4 (COX4), a key regulatory subunit of mammalian cytochrome c oxidase, could promote these effects. To understand how the two COX4 isoforms (COX4-1 and COX4-2) differentially affect glioma metabolism, glioma samples harvested from COX4-1- or COX4-2-overexpressing U251 cells were profiled using Gas chromatography–mass spectrometry GC-MS and Liquid Chromatography - Tandem Mass Spectrometry LC-MS/MS metabolomics platforms. The concentration of 362 metabolites differed significantly in the two cell types. The two most significantly upregulated pathways associated with COX4-1 overexpression were purine and glutathione metabolism; the two most significantly downregulated metabolic pathways associated with COX4-1 expression were glycolysis and fatty acid metabolism. Our study provides new insights into how Cytochrome c oxidase (CcO) regulatory subunits affect cellular metabolic networks in GBM and identifies potential targets that may be exploited for therapeutic benefit.

## 1. Introduction

Cytochrome c oxidase (CcO), which is present in all prokaryotes and eukaryotes, is a critical regulator of oxidative phosphorylation (OXPHOS) and ATP generation [1,2]. As the terminal enzyme in the mitochondrial electron transport chain, CcO catalyzes the transfer of electrons from cytochrome c to oxygen, which produces water and the proton gradient necessary for ATP production and for the exchange of metabolites and ions between the mitochondria and the cytosol and other organelles [3]. In this capacity, CcO is an essential enzyme, as evidenced by the plethora of pathologic conditions associated with CcO deficiency, including hearing and visual impairments, exercise intolerance, encephalomyopathy, neuropathy, and cardiomyopathy, among others [3,4,5]. Emerging evidence now suggests a crucial role for CcO in regulating tumorigenesis and the response to treatment in various cancers as well [2,6,7,8,9,10,11,12], although the molecular mechanisms involved have not been well defined.

The mammalian CcO enzyme is composed of 14 subunits encoded by either the mitochondrial or the nuclear genome [6,13,14]. The three mitochondrial-encoded CcO subunits (COX1, COX2, and COX3) constitute the catalytic core of the enzyme, whereas the smaller nuclear-encoded subunits are peripheral to the catalytic core and appear to be involved in the regulation and stability of the fully assembled enzyme complex [1,15]. We and others have now shown that the isoform expression of specific nuclear-encoded CcO subunits is associated with aggressive malignant behavior and resistance to apoptosis in breast carcinoma (COX7AR), lung adenocarcinoma (COX6B2), and glioblastoma (GBM) (COX4) [9,10,11,12,16,17,18] cells.

COX4, which is the largest regulatory subunit of CcO, controls the enzymatic activity of CcO in response to cellular ATP concentrations [19], enhancing the activity of CcO and, thus, OXPHOS when ATP levels are low [20]. Two isoforms of COX4 (COX4-1 and COX4-2) have been detected in mammals [21]; the expression of COX4-1 is required for CcO to couple the rate of ATP production to energetic requirements. Although these isoforms are highly homologous in the C-terminal region, the N-terminal regions of COX4-1 and COX4-2 contain crucial differences. In particular, COX4-2 lacks the serine 58 residue that regulates ATP binding [1,20] and contains three cysteine residues that appear to serve as redox sensors [21]. Our studies of tumor specimens from patients with GBM further demonstrated that high tumor expression of COX4-1 correlates in a significant manner with poor patient prognosis, whereas high COX4-2 expression does not, suggesting novel functions for these COX4 isoforms [16]. Additionally, we confirmed that a transition from COX4-2 to COX4-1 expression triggers a decrease in the concentration of superoxide and the onset of radio- and chemoresistance in U251 glioma cells.

In the present study, we used an untargeted metabolomic profiling platform to quantify biochemical differences within the glioma cells overexpressing the individual COX4 isoforms used in our previous studies [12,16]. We show that stable overexpression of the COX4-1 isoform is associated with many metabolic alterations.

## 2. Materials and Methods

### 2.1. Glioma Cell Lines

Human glioma cell line U251 was originally obtained from Dr. G. Yancey Gillespie (University of Alabama at Birmingham, Birmingham, AL, USA) and was authenticated using the short tandem repeat (ATCC, STR service, Manassas, VA, USA). Cells were grown as we previously described [1,2,3] in DMEM F-12 medium plus L-glutamine supplemented with 7% heat-inactivated FBS, penicillin, and streptomycin. Cells were incubated at 37 °C in a humidified atmosphere containing 5% CO_2_. The generation of COX4-1- and COX4-2-overexpressing cells was previously described [3,4,5]. Briefly, U251 COX4-2-knockout (COX4-2-KO) cells were generated using a CompoZr Knockout ZFN Kit (Sigma-Aldrich, St. Louis, MO, USA) according to manufacturer instructions. U251 COX4-2-KO cells were electroporated with CMV6 plasmids containing FLAG-epitope-tagged COX4-2 or COX4-1 (Catalog # RC209204 and RC209374, OriGene Technologies, Rockville, MD, USA). All Electroporation were performed using a Gene Pulser Xcell Electroporation System (BioRad, Hercules, CA, USA) using the following conditions: square wave pulse, 25 ms, and 140 V. To generate stable cell lines overexpressing COX4-1 or COX4-2, cells were selected with G418 for 2 weeks. The stable lines isolated were characterized for the level of mitochondrial COX4-1 and COX4-2 by Western blot analysis [3].

### 2.2. Sample Preparation

Samples were prepared using the automated MicroLab STAR system (Hamilton, Reno, NV, USA). Several recovery standards were added prior to the first step in the extraction process for quality control purposes. Proteins were precipitated with methanol under vigorous shaking followed by centrifugation. The resulting extract was divided into five fractions: two for analysis by two separate reversed-phase ultrahigh performance LC (RP/UPLC)-MS/MS methods with positive ion mode electrospray ionization (ESI), one for analysis by RP/UPLC-MS/MS with negative ion mode ESI, one for analysis by hydrophilic interaction LC (HILIC)/UPLC-MS/MS with negative ion mode ESI, and one sample for a backup. Samples were placed briefly on a TurboVap (Zymark Corporation, Hopkinton, MA, USA) to remove the organic solvent. The sample extracts were stored overnight under nitrogen before preparation for analysis.

Several types of controls were analyzed in concert with the experimental samples: a pooled matrix sample generated by taking a small volume of each experimental sample served as a technical replicate throughout the data set; extracted water samples served as process blanks; and a cocktail of QC standards that were carefully chosen not to interfere with the measurement of endogenous compounds was spiked into every analyzed sample to allow instrument performance monitoring and aid chromatographic alignment. Instrument variability was determined by calculating the median relative standard deviation (RSD) for the standards that were added to each sample prior to injection into the mass spectrometers. Overall process variability was determined by calculating the median RSD for all endogenous metabolites (i.e., non-instrument standards) present in 100% of the pooled matrix samples. Experimental samples were randomized across the platform and run with QC samples spaced evenly among the injections.

### 2.3. UPLC-MS/MS

All methods were performed with a Waters ACQUITY UPLC system and a Thermo Scientific Q-Exactive high-resolution/accurate mass spectrometer interfaced with a heated electrospray ionization (HESI-II) source and Orbitrap mass analyzer operated at 35,000 mass resolution. The sample extract was dried, then reconstituted in solvents compatible with each for the four methods. Each reconstitution solvent contained a series of standards at fixed concentrations to ensure injection and chromatographic consistency. One aliquot was analyzed using acidic positive ion conditions, chromatographically optimized for more hydrophilic compounds. In this method, the extract was gradient eluted from a C18 column (Waters UPLC BEH C18-2.1 × 100 mm, 1.7 µm) using water and methanol, containing 0.05% perfluoropentanoic acid (PFPA) and 0.1% formic acid (FA). Another aliquot was also analyzed using acidic positive ion conditions but was chromatographically optimized for more hydrophobic compounds. In this method, the extract was gradient eluted from the same aforementioned C18 column using methanol, acetonitrile, water, 0.05% PFPA, and 0.01% FA and was operated at an overall higher organic content. Another aliquot was analyzed using basic negative-ion-optimized conditions and eluted with a separate dedicated C18 column. The basic extracts were gradient eluted from the column using methanol and water, but with 6.5 mM ammonium bicarbonate at pH 8. The fourth aliquot was analyzed via negative ionization following elution from an HILIC column (Waters UPLC BEH Amide 2.1 × 150 mm, 1.7 µm) using a gradient consisting of water and acetonitrile with 10 mM ammonium formate, pH 10.8. The MS analysis alternated between MS and data-dependent MS^n^ scans using dynamic exclusion. The scan range varied slightly between methods but covered 70–1000 *m*/*z*.

### 2.4. Bioinformatics

The informatics system consisted of four major components, the Laboratory Information Management System (LIMS), the data extraction and peak-identification software, data processing tools for QC and compound identification, and a collection of information interpretation and visualization tools for use by data analysts. The hardware and software foundations for these informatics components were the LAN backbone and a database server running Oracle 10.2.0.1 Enterprise Edition.

### 2.5. Data Extraction and Compound Identification

Raw data were extracted, peak identified, and QC processed using Metabolon’s hardware and software. These systems were built on a web-service platform utilizing Microsoft. NET technologies run on high-performance application servers and fiber-channel storage arrays in clusters to provide active failover and load-balancing. Compounds were identified by comparison to library entries of purified standards or recurrent unknown entities. Metabolon maintains a library based on authenticated standards that contains the retention time/index (RI), mass-to-charge ratio (*m/z)*, and chromatographic data (including MS/MS spectral data) on all molecules present in the library. Furthermore, biochemical identifications are based on three criteria: retention index within a narrow RI window of the proposed identification, accurate mass match to the library ±10 ppm, and the MS/MS forward and reverse scores between the experimental data and authentic standards. The MS/MS scores are based on a comparison of the ions present in the experimental spectrum to the ions present in the library spectrum. While there may be similarities between these molecules based on one of these factors, the use of all three data points can be utilized to distinguish and differentiate biochemicals. More than 3300 commercially available purified standard compounds were acquired and registered into LIMS for analysis on all platforms for determination of their analytical characteristics.

### 2.6. Metabolite Quantification and Data Normalization

Peaks were quantified by area-under-the-curve integration and normalized to total protein as determined by Bradford assay to account for differences in metabolite levels due to differences in the amount of material present in each sample.

### 2.7. Statistical Analysis

Two types of statistical analysis were performed: (1) significance tests and (2) classification analysis. Standard statistical analyses were performed in ArrayStudio on log-transformed data. For those analyses not standard in ArrayStudio, the program R (http://cran.r-project.org/ (accessed on 14 June 2022)) or the program JMP was used.

## 3. Results

### 3.1. Effects of COX4 Isoforms on the Glioma Cell Metabolome

**Figure 1:**
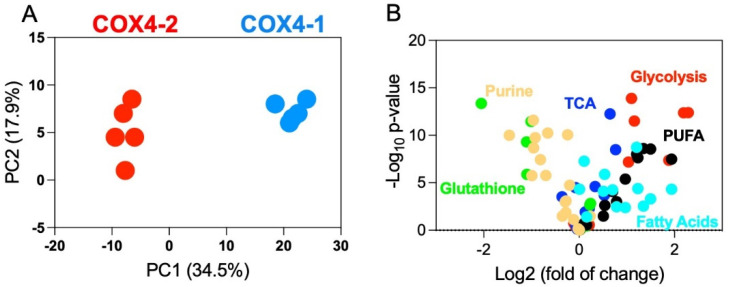
Metabolite profiling of U251 glioma cells overexpressing the COX4-1 or COX4-2 isoform. (**A**) Two-dimensional PCA score plots of untargeted metabolomics data from COX4-1-overexpressing glioma cells (blue) and COX4-2-overexpressing glioma cells (red). (**B**) Volcano plot of metabolomic data. The x-axis represents the mean fold change (log_2_ ratio) in the relative intensity of each metabolite between the two samples (COX4-2-overexpressing relative to the COX4-1-overexpressing cells). The y-axis represents the statistical significance (-log_10_-transformed *p*-values) of each metabolite. Metabolites are color coded by metabolic pathway.

Using an integrated metabolomics platform [6], we identified 519 compounds of known identity (biochemicals) in U251 cells overexpressing COX4-1 or COX4-2 (n = 5/isoform) [3,4,5]. Analysis by two-way ANOVA identified 362 biochemicals that achieved statistical significance (*p* ≤ 0.05) and exhibited significant interaction and main effects for experimental parameters of genotype. Among those biochemicals, 183 biochemicals were upregulated and 179 biochemicals were downregulated in the COX4-1-overexpressing cells compared with the levels in COX4-2-overexpressing cells. Principal component analysis (PCA), an unsupervised multivariate statistical method, showed a clear statistically significant distinction between the COX4-1- and COX4-2-overexpressing cells (Figure 1A). The two principal components, PC1 and PC2, accounted for 34.5% and 17.9% of the total data variation at baseline.

To identify the metabolites driving the distinction between the two isoform-overexpressing cell types, we performed an ANOVA to compare the relative levels of individual metabolites and altered common pathways in each. The two most significantly upregulated pathways associated with COX4-1 overexpression were purine and glutathione metabolism; the two most significantly downregulated metabolic pathways associated with COX4-1 overexpression were glycolysis and fatty acid metabolism (Figure 1B, complete data provided in Supplementary Table S1). These findings indicate that changes in the glioma cell metabolome correlate with a change in the COX4 isoform expressed, prompting us to further examine the effect of COX4 isoform expression on the metabolites involved in these four pathways.

### 3.2. Analysis of Glucose Metabolism in Glioma Cells Overexpressing COX4 Isoforms

**Figure 2:**
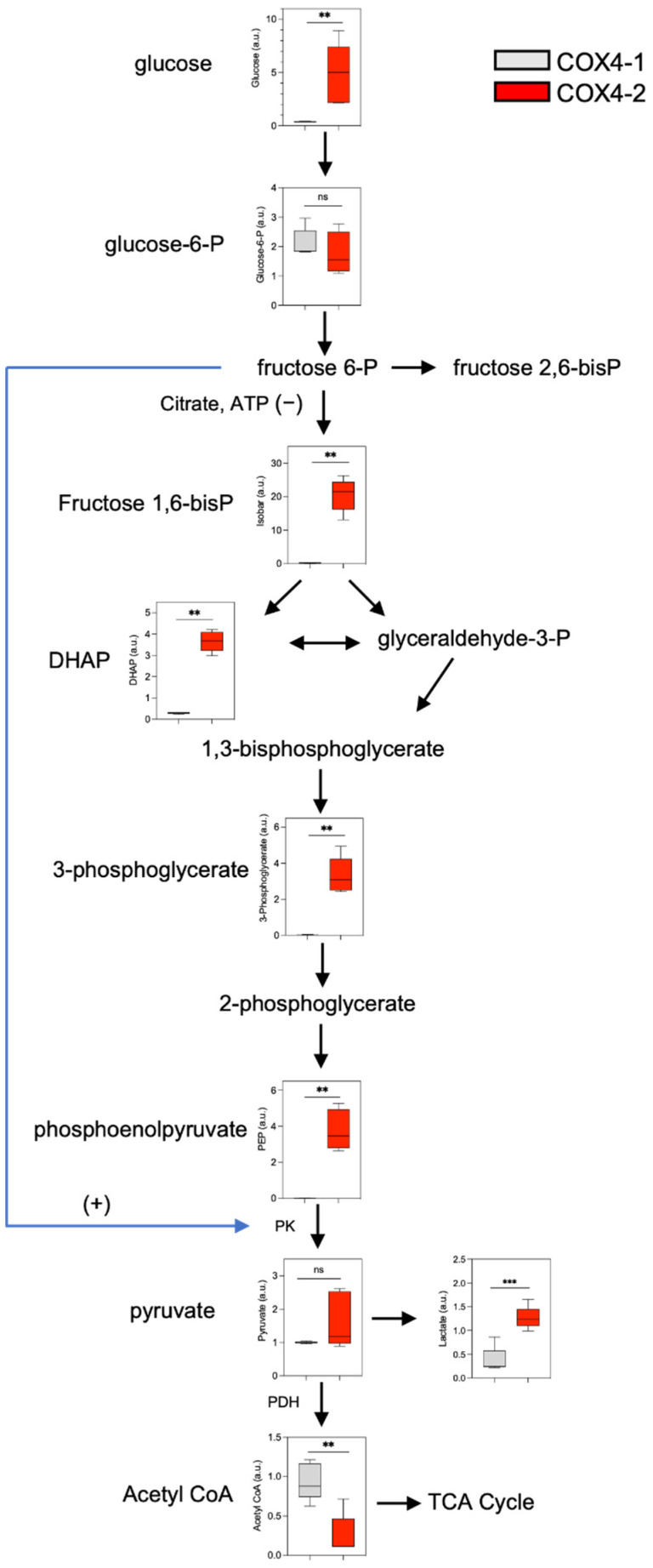
Effects of COX4 isoform expression on glycolysis metabolites in glioma cells. Boxplots depict the levels of key metabolites involved in glycolysis analyzed in COX4-1-(gray boxes) and COX4-2 (red boxes)-overexpressing cells. The horizontal line inside the box indicates the median value. Results of five independent measurements. **, *p* < 0.01; ***, *p* < 0.001; a.u., arbitrary units; ns, not significant.

The levels of most glycolysis pathway metabolites were downregulated in the COX4-1-overexpressing glioma cells relative to those in the COX4-2-overexpressing cells. Glucose 6-phosphate levels were similar in the two cell groups, but the levels of glucose (13-fold), dihydroxyacetone phosphate (DHAP), fructose 6-phosphate, fructose 1,6-bisphosphate (isobar), 3-phosphoglycerate, and phosphoenolpyruvate (PEP) were, respectively, 12-fold, 76-fold, 157-fold, and 199-fold lower in the COX4-1-overexpressing cells. Curiously, however, pyruvate levels were not greatly influenced by the COX4 isoform expressed (Figure 2).

We hypothesized that the lower levels of glycolysis metabolites downstream of glucose-6-phosphate detected in the COX4-1-overexpressing cells may reflect an increase in the conversion of pyruvate to acetyl-CoA for entry into the TCA cycle, which produces the reducing equivalents NADH and FADH_2_ that support electron transport in OXPHOS [7,8]. In line with this possibility, the steady-state level of acetyl-CoA was four-fold higher in the COX4-1-overexpressing cells than in the COX4-2-overexpressing cells, while the level of lactate was three-fold higher in the COX4-2-overexpressing cells than in the COX4-1-overexpressing cells (Figure 2). In addition, the level of glutamine, which can undergo glutaminolysis to produce α-ketoglutarate (αKG) and, thus, support TCA cycle metabolism, was two-fold lower in the COX4-1-overexpressing cells. A lower level of glutamine would be consistent with a greater dependence on glutaminolysis to support the TCA cycle.

**Figure 3:**
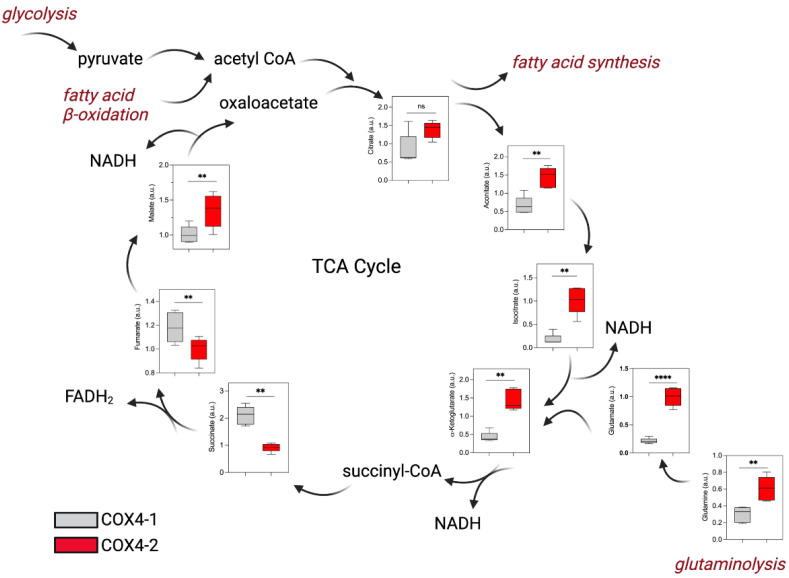
Effects of COX4 isoform expression on TCA cycle metabolites in glioma cells. Boxplots depict the levels of key metabolites involved in the TCA cycle analyzed in COX4-1- and COX4-2-overexpressing cells. The horizontal line inside the box indicates the median value. Results of five independent measurements. **, *p* < 0.01; ****, *p* < 0.0001; a.u., arbitrary units; ns, not significant.

Lower levels of citrate (2-fold), aconitate (2-fold), isocitrate (6-fold), and αKG (3.5-fold) were also detected in the COX4-1-overexpressing cells and may be consistent with increased consumption of citrate for fatty acid synthesis (Figure 3). Conversely, the levels of fumarate and succinate, two TCA metabolites associated with remodeling of the cancer epigenome [9,10], were significantly higher in the COX4-1-overexpressing cells. Although questions remain about the influences on specific metabolites, these data strongly indicate that COX4 isoform expression influences glucose metabolism in glioma cells.

### 3.3. Analysis of Fatty Acid Metabolism in Glioma Cells Overexpressing COX4 Isoforms

**Figure 4:**
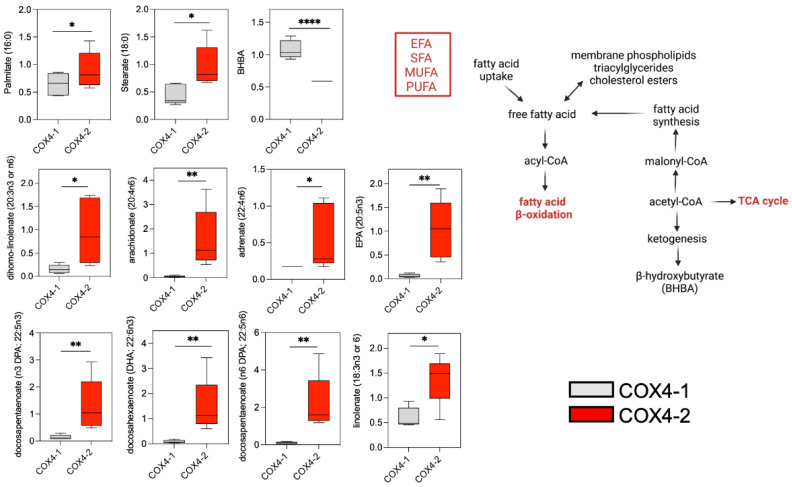
Effects of COX4 isoform expression on fatty acid pathway metabolites in glioma cells. Boxplots depict the levels of key metabolites involved in fatty acid metabolism analyzed in COX4-1- and COX4-2-overexpressing cells. The horizontal line inside the box indicates the median value. Results of five independent measurements. *, *p* < 0.05; **, *p* < 0.01; ****, *p* < 0.0001; a.u., arbitrary units; ns, not significant; EFA, essential fatty acids; SFA, saturated fatty acids; MUFA, monounsaturated fatty acids. (Right) Schematic representation of fatty acid metabolism.

Fatty acid β-oxidation generates acetyl-CoA for entry into the TCA cycle to support OXPHOS and, thereby, cell proliferation, and this pathway is upregulated in at least some cancer cells [11,12]. In conjunction with the high levels of acetyl-CoA in the COX4-1-overexpressing cells (Figure 2), many free fatty acids (FFA), including palmitate (1.5-fold) and stearate (2.5-fold), were markedly reduced in COX4-1-overexpressing cells relative to COX4-2-overexpressing cells. The levels of polyunsaturated fatty acids (PUFA) were particularly altered by COX4-1 overexpression. Among n-6 PUFA, the levels of dihomo-linolenate (6-fold), arachidonate (32-fold), adrenate (3-fold), and docosapentaenoate (24-fold) were significantly reduced in COX4-1 cells. Among n-3 PUFA, eicosapentaenoic acid (EPA; 15-fold), linolenate (2-fold), docosapentaenoate (10-fold), and docohexaenoate (17-fold) were significantly reduced in COX4-1 cells. In addition, upregulation of 3-hydroxybutyrate (BHBA, 2-fold), which could reflect excess acetyl-CoA production [13], was detected in the COX4-1-overexpressing cells (Figure 4). Overall, these results are consistent with COX4 isoform expression regulating FFA β-oxidation and, consequently, the availability of acetyl-CoA for entry into the TCA cycle in glioma cells.

### 3.4. Analysis of Glutathione Metabolism in Glioma Cells Overexpressing COX4 Isoforms

**Figure 5:**
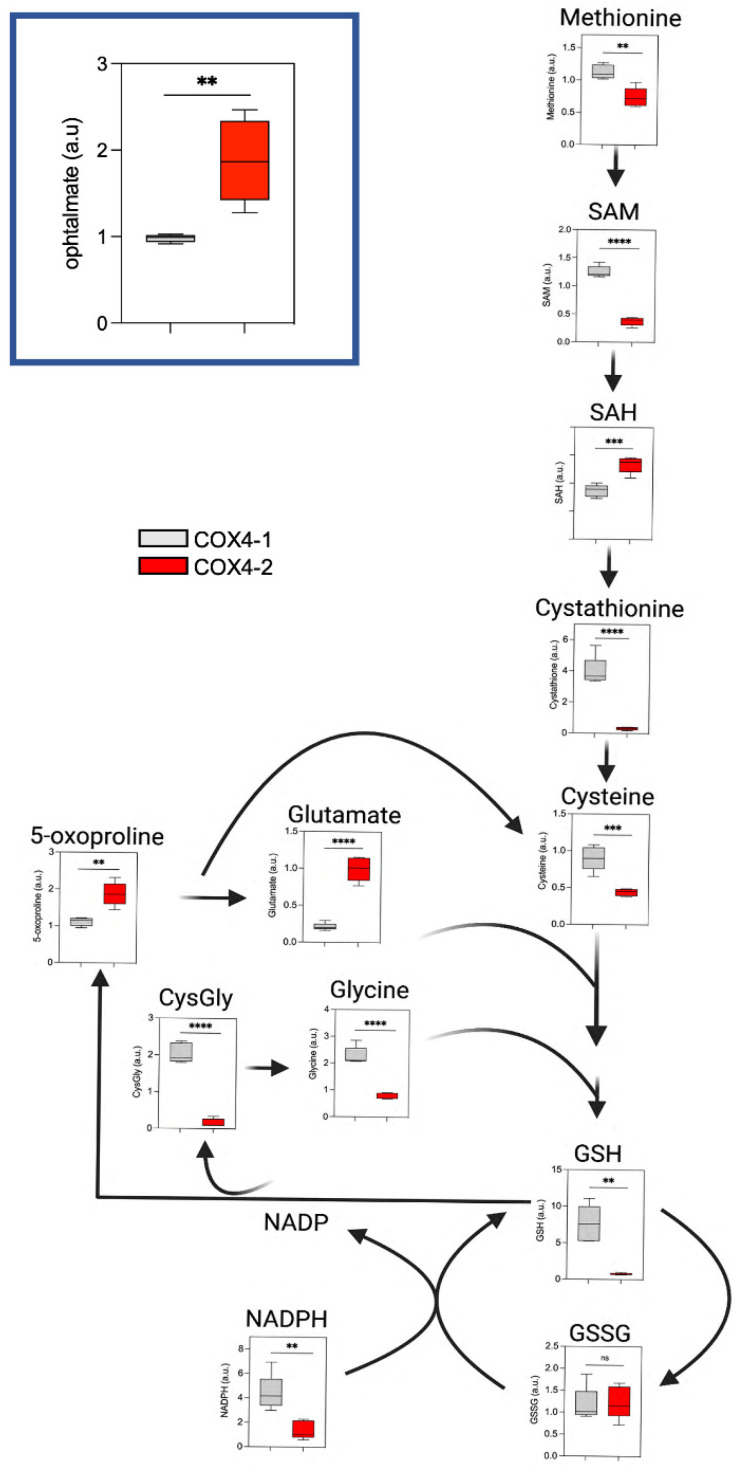
Effects of COX4 isoform expression on glutathione pathway metabolites in glioma cells. Boxplots depict the levels of key metabolites involved in the glutathione pathway analyzed in COX4-1- and COX4-2-overexpressing cells. The horizontal line inside the box indicates the median value. Results of five independent measurements. **, *p* < 0.01; ***, *p* < 0.001; ****, *p* < 0.0001; a.u., arbitrary units; ns, not significant.

Glutathione metabolism is critically involved in maintaining the redox balance in cells and dysregulation of this pathway has been linked to cell growth and resistance to treatment in GBM and other cancers [14,15,16,17]. Redox stress in cells can be, at least in part, reflected by the ratio of reduced glutathione (GSH) to oxidized glutathione (GSSG), with a higher ratio indicative of reduced oxidative stress. Glutathione is synthesized from cysteine, with cysteine being the limiting component. To meet increased demand for glutathione, cells can supplement the uptake of extracellular cysteine with intracellular synthesis from methionine via the transsulfuration pathway [18] (Figure 5). Metabolites involved in this process, including cysteine (2-fold), methionine (1.5-fold), and GSH (10-fold), were upregulated in the COX4-1-overexpressing glioma cells (Figure 5), suggesting that the transsulfuration pathway is more active in cells expressing the COX4-1 isoform than in cells expressing COX4-2. In line with this, the concentration of 5-oxoproline, a metabolite involved in the glutathione salvage pathway, was two-fold higher in COX4-2-overexpressing cells. Finally, the levels of ophthalmate, which have been shown to inversely correlate with GSH depletion and oxidative stress [19], were two-fold lower in the COX4-1-overexpressing cells than in the COX4-2-overexpressing cells (Figure 5, inset). These results indicate that the COX4 isoforms differentially regulate glutathione metabolism and, thereby, the resistance to oxidative stress in glioma cells.

### 3.5. Analysis of Purine Nucleotide Synthesis in Glioma Cells Overexpressing COX4 Isoforms

**Figure 6:**
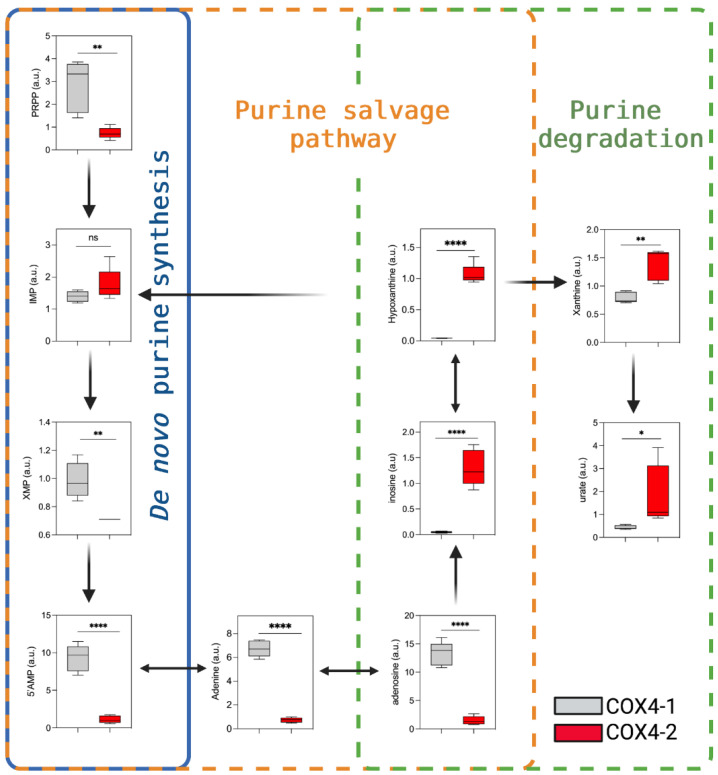
Effects of COX4 isoform expression on purine metabolism in glioma cells. Boxplots depict the levels of key metabolites involved in purine metabolism analyzed in COX4-1- and COX4-2-overexpressing cells. The horizontal line inside the box indicates the median value. Results of five independent measurements. *, *p* < 0.05; **, *p* < 0.01; ****, *p* < 0.0001; a.u., arbitrary units; ns, not significant; PRPP, phosphoribosyl-1-pyrophosphate.

A growing body of literature indicates that purine synthesis contributes to the aggressive behavior of GBM and other cancers [20,21]. The levels of metabolites involved in the de novo synthesis of purines were higher in COX4-1-overexpressing cells than in COX4-2-overexpressing cells. Specifically, phosphoribosyl pyrophosphate (PRPP), xanthosine 5′-monophosphate (XMP), and 5′AMP were 4-fold, 1.5-fold, and 8.4-fold higher, respectively, in COX4-1-overexpressing cells. Additionally, the level of adenosine and adenine, metabolites involved in the purine salvage pathway, were 8.6-fold and 9-fold higher in COX4-1-overexpressing cells (Figure 6). Interestingly, metabolites involved in the purine degradation pathway were significantly higher in COX4-2-overexpressing cells, including hypoxanthine (25-fold), inosine (26-fold), xanthine (2-fold), and urate (4.3-fold). These results suggest the mechanisms by which the COX4 isoforms could differentially regulate purine metabolism in glioma cells.

## 4. Discussion

CcO is an important mitochondrial complex required for the regulation of mitochondrial OXPHOS and ATP generation [22,23]. Although the functions in mammalian organisms of the mitochondrial-encoded CcO subunits are well established, relatively less is known about the role of the nuclear-encoded regulatory subunits in the assembly and activity of the complex [24]. We previously demonstrated that expression of the COX4-1 isoform is associated with elevated CcO activity and chemoresistance in GBM cells [1,2,3]. Additionally, using an in vitro model of GBM adaptive radioresistance, we found that exposure of glioma cells to fractionated radiation promotes a switch from the expression of the COX4-2 isoform to the COX4-1 isoform, which, in turn, promotes the assembly of mitochondrial supercomplexes that enhance OXPHOS while minimizing ROS production [5]. In agreement with our results, it was recently reported that deletion of the COX4-1 subunit in HEK293 results in complete CcO deficiency and disruption of supercomplex assembly [25]. Here, we report that the COX4 isoforms differentially modulate the metabolic profile of glioma cells, in particular, the concentration of metabolites involved in glycolysis, fatty acid metabolism, purine metabolism, and glutathione metabolism.

Although glioma cells preferentially utilize glycolysis as the primary source of energy production, as originally postulated by Warburg [26,27], our previous studies showed that OXPHOS, mitochondrial respiration, and ATP production are upregulated in glioma cells expressing COX4-1 instead of COX4-2. Results from this metabolomic analysis now reveal that COX4-1 overexpression modulates the steady-state levels of glycolysis and TCA-cycle intermediates relative to levels in COX4-2-overexpressing cells. In particular, we detected a clear signature of reduced glucose uptake and utilization in the COX4-1-overexpressing cells.

It is possible that the decreased levels of glycolysis metabolites in the COX4-1-overexpressing cells reflect the increased production of acetyl-CoA, which could then feed into the TCA cycle to generate the NADH and FADH_2_ necessary to support an increase in OXPHOS. The lower levels of citrate, aconitate, isocitrate, and αKG may reflect this possibility, although it is also possible that the low levels of these metabolites instead reflect the reverse pathway, in which acetyl-CoA is produced from αKG to support fatty acid synthesis. The latter possibility fits with the evidence that the conversion of glutamine to αKG and subsequent production of citrate supports fatty acid synthesis in cancer cells with dysregulated TCA-cycle products [28]. There is a TCA-cycle-independent cytoplasmic pathway of reductive carboxylation of αKG, which is mediated by IDH1 [29]. This pathway results in the formation of isocitrate, citrate, and, finally, in acetyl-CoA for lipid synthesis [8].

The COX4-1-associated differences in succinate and fumarate levels are particularly intriguing. These metabolites are structurally similar to αKG and competitively inhibit multiple αKG-dependent dioxygenases, leading to alterations in genome-wide histone and DNA methylation [10]. We previously showed, in orthotopic mouse models, that overexpression of COX4-1 in glioma cells promotes the development of invasive tumors, characterized by multiple tumor loci throughout the entire brain parenchyma. No tumors were detected in the brains from mice inoculated with COX4-2-overexpressing cells, indicating a significantly slower progression of these tumors in vivo [3]. Since αKG-dependent dioxygenases have been involved in the regulation of cell growth in several cancer cells [30,31,32,33], it is possible that COX4-1 overexpression stimulates glioma cell growth through the upregulation of succinate and fumarate.

Our results further indicate that the COX4 isoforms differentially affect fatty acid β-oxidation. Supporting this finding, expression profiling of GBM previously revealed increased expression of genes associated with lipid and phospholipid uptake and catabolism, indicating a phenotype of lipid utilization [34,35]. Moreover, fatty acid oxidation is a key driver of progression from low-grade glioma to GBM [35]. Our study suggests that when COX4-1 is overexpressed, GBM cells adapt to support the associated increase in growth and proliferation by partially shifting their metabolism toward fatty acid oxidation. Oxidation of fatty acids enhances not only the production of acetyl-CoA and, thus, of ATP, but also the production of the reducing equivalents for anabolic processes, all of which support cancer cell proliferation [11,12].

We also present the novel observation that glioma cells overexpressing the COX4-1 isoform produce the ketone body BHBA. Ketogenesis generally occurs when glucose levels drop after fasting and has been detected primarily in hepatocytes, but also in kidney and intestinal epithelial cells as well as astrocytes, which possess the proper enzymatic machinery [13,36]. Although unexpected, especially as previous research revealed that most cancer cells do not express the enzymes necessary to utilize ketone bodies [37,38,39,40,41], the up regulation of BHBA observed in COX4-1-overexpressing cells is not entirely without precedent. Grabacke et al. [36] previously reported that murine melanoma (B16 F10) cells and human GBM (LN-229) cells are capable of efficient synthesis and release of BHBA when treated with a PPARa agonist, although the induced ketogenesis seems to be independent of PPARa expression or activity in those cells. However, a role for COX4 in BHBA production has not been described and the underlying mechanism remains to be determined.

Interestingly, increased fatty acid oxidation has also been associated with higher resistance to radio- and chemotherapy in cancer cells [42,43]. The low levels of PUFA we detected in COX4-1-overexpressing cells may also contribute to the development of treatment resistance in GBM. Recent studies have shown that radiation-induced cancer cell death relies, in part, on ferroptosis induced by lipid peroxidation [44,45]. Our previous study further showed that the switch from COX4-2 to COX4-1 expression, induced by fractionated radiation, paradoxically reduces lipid peroxidation in glioma cells, ultimately promoting radioresistance [4]. As ferroptosis appears to rely in large part on the peroxidation of PUFAs, specifically [44,45], the reduced availability of PUFAs in COX4-1-overexpressing cells detected in this metabolomic study may explain the lack of lipid peroxidation.

We previously showed that expression of COX4-1 in glioma cells also correlates with alterations consistent with reduced oxidative stress, including a reduction in the level of superoxide and an increase in the level of the powerful antioxidant GSH [1,2,4,5]. In agreement with these previous results, the level of GSH was higher in COX4-1-overexpressing glioma cells than in COX4-2-overexpressing glioma cells. De novo synthesis of GSH relies on the availability of cysteine, which can be replenished via extracellular import by the cystine–glutamate antiporter xCT or via intracellular synthesis through the transsulfuration pathway [46]. Both routes are important for maintaining the antioxidant potential in cells under oxidative stress when the demand for GSH is elevated [46,47]. Our results presented here suggest that COX4-1-overexpressing glioma cells rely, at least in part, on the transsulfuration pathway to support the increase in GSH synthesis.

Interestingly, we found that the levels of ophthalmate were significantly elevated in COX4-2-overexpressing glioma cells. Ophthalmate is a GSH analog, the synthesis of which is mediated by the same enzymes that produce GSH but involves different metabolites, depriving it of the reducing properties in GSH [48,49,50]. Evidence suggests that ophthalmate can be considered a marker of GSH depletion, as ophthalmate levels increase when the availability of cysteine is limited and GSH levels decrease [19,51,52]. Therefore, the relatively low level of ophthalmate in COX4-1-overexpressing cells further suggests an increase in GSH. Oxidative stress has long been implicated in cancer development and progression [53]. Studies in a variety of tumor cell types, including GBM, have suggested that cancer chemotherapy drugs induce apoptosis, in part, by enhancing oxidative stress [1,2,54] and increasing evidence demonstrates that GSH has a prominent role in resistance to chemotherapy [55,56,57]. Therefore, our results suggest that COX4-1-induced resistance to chemotherapy is mediated, in part, by upregulation of the transsulfuration pathway and the consequent overproduction of GSH.

Finally, a growing body of literature indicates that the synthesis of purines contributes to the aggressive behavior and therapy resistance of GBM [20,21,58,59]. Here, we showed that overexpression of COX4-1 is associated with high levels of GMP, xanthosine 5′-monophosphate, and AMP. These biochemicals serve as essential building blocks for DNA replication and for RNA production to support protein synthesis and to sustain proliferative capacity. As we recently showed, expression of COX4-1 is associated with enhanced ability to repair radiation-induced DNA damage in glioma cells [4,5], suggesting that the high rates of de novo purine synthesis in COX4-1-overexpressing glioma cells may contribute to the more aggressive and radioresistant phenotype observed upon the switch from COX4-2 to COX4-1 expression.

## 5. Conclusions

The present study extends and supports our previous findings in glioma cells, identifying several key metabolic pathways that are affected by the CcO regulatory subunit COX4 isoform expressed and may underlie the clinical progression and development of therapeutic resistance in GBM. Although the changes in metabolites detected in our analyses may also be products or substrates in cellular pathways other than those we discussed, the metabolic profiles described agree with those in our recent publications, in which we characterized the bioenergetic profile of COX4-1- and COX4-2-expressing glioma cells [2,3,4,5]. The seemingly conflicting effects of COX4-1 overexpression on some metabolites within these pathways indicate the high degree of complexity involved, however, and point to the need for more intricate studies to fully understand how these pathways interact to promote GBM aggression and resistance to therapy.

## Data Availability

The data presented in this study are available in article and Supplementary Material.

## Supplementary Materials

The following supporting information can be downloaded at: https://www.mdpi.com/article/10.3390/metabo12080748/s1, Table S1: Metabolite profiling of U251 glioma cells overexpressing the COX4-1 or COX4-2 isoform.
